# Supplementary material for: Histological Analysis of Gonadal Ridge Development and Sex Differentiation of Gonads in Three Gecko Species
Source: Biology (Basel). 2023 Dec 22;13(1):7. doi: 10.3390/biology13010007 (PMC10813461; doi:10.3390/biology13010007)
Supplement: Supplementary file 1 [file biology-13-00007-s001.zip › biology-2690443-supplementary.pdf]

Suppl. Table S1. Number of specimen analysed.

Stages	Days of incubation	Specimen with undifferentiated gonads	Specimen with ovaries	Specimen with testes	Sum of specimen studied
Crested gecko (*Correlophus ciliatus*)					
S29	D5	2	0	0	2
S30	D6	3	0	0	3
S31	D7	2	0	0	2
S32	D8	3	0	0	3
S32	D9	4	0	0	4
S32	D10	1	3	2	6
S34	D15	0	2	3	5
S35	D17	0	0	1	1
S35	D18	0	3	2	5
S36	D22	0	0	1	1
S36	D25	0	2	2	4
Leopard gecko (*Eublepharis macularius*) 26°C – female producing temperature (FPT)					
S29	D5	1	0	0	1
S30	D6	2	0	0	2
S31	D10	2	0	0	2
S32	D15	1	0	0	1
S33	D20	0	2	1	3
S35	D26	0	2	0	2
S37	D30	0	3	0	3
Leopard gecko (*Eublepharis macularius*) 32°C – male producing temperature (MPT)					
S29	D4	1	0	0	1
S30	D5	2	0	0	2
S31	D7	2	0	0	2
S32	D10	1	0	2	3
S34	D15	0	0	2	2
S35	D20	0	0	1	1
S37	D27	0	1	1	2
S41	D35	0	0	2	2
Mourning gecko (*Lepidodactylus lugubris*)					
S29	D7	2	0	0	2
S30	D10	1	0	0	1
S31	D20	2	1	0	3
S32	D25	0	3	0	3
S33	D30	0	3	0	3
S34	D40	0	3	0	3
S36	D50	0	2	0	2
S38	D55	0	2	0	2

Suppl. Table S2. Measurements of sizes and germ cell number in gonads at the stage of sex differentiation.

Measurement	Crested gecko (*Correlophus ciliatus*) N=6	Leopard gecko (*Eublepharis macularius*) N=6	Mourning gecko (*Lepidodactylus lugubris*) N=3
Mean thickness of the coelomic epithelium covering the gonadal ridge	8.78 ± 2.74 µm	8.58 ± 3.03 µm	9.82 ± 2.95 µm
Mean thickness of the coelomic epithelium of the dorsal mesentery	4.13 ± 1.66 µm[a]	2.70 ± 0.97 µm[a]	3.81 ± 1.01 µm[a]
Mean thickness of the cortex in the differentiating ovaries	36.4 ± 6.8 µm	35.2 ± 5.4 µm	20.5 ± 4.4 µm
Mean thickness of the cortex in the differentiating testes	15.6 ± 5.4 µm[a]	19.8 ± 4.8 µm[a]	-
Mean diameter of the medulla in the differentiating ovaries	65.3 ± 8.7 µm	107.4 ± 12.3 µm	64.3 ± 9.2 µm
Mean diameter of the medulla in the differentiating testes	66.9 ± 7.5 µm	71.2 ± 8.2 µm[a]	-
Mean number of germ cells per cross-section of the cortex of the differentiating ovaries	3.3 ± 0.7	5.2 ± 2.2	4.6 ± 1.2
Mean number of germ cells per cross-section in the cortex of the differentiating testes	0.9 ± 0.9[a]	0.7 ± 0.7[a]	-
Mean number of germ cells per cross-section in the medulla of the differentiating ovaries	0.8 ± 0.8	0.2 ± 0.4	1.2 ± 0.6
Mean number of germ cells per cross-section in the medulla of the differentiating testes	2.8 ± 0.9[a]	2.4 ± 1.1[a]	-

[a] Significant difference between ovaries and testes (χ^2 test, P<0.05).
